# Systemic arterial hypertension leads to decreased semen quality and alterations in the testicular microcirculation in rats

**DOI:** 10.1038/s41598-019-47157-w

**Published:** 2019-07-30

**Authors:** Lucas Giglio Colli, Larissa Berloffa Belardin, Cinthya Echem, Eliana Hiromi Akamine, Mariana Pereira Antoniassi, Rhayza Roberta Andretta, Lucas Solla Mathias, Stephen Fernandes de Paula Rodrigues, Ricardo Pimenta Bertolla, Maria Helena Catelli de Carvalho

**Affiliations:** 1 Department of Pharmacology, Division of Vascular Biology, Hypertension Section, Instituto de Ciências Biomédicas, Universidade de São Paulo –ICB/USP, São Paulo, SP Brazil; 2 Department of Surgery, Division of Urology, Universidade Federal de São Paulo - UNIFESP, São Paulo, Brazil; 3 Department of Internal Medicine, Botucatu Medical University, Universidade Estadual Paulista - UNESP, Botucatu, São Paulo Brazil

**Keywords:** Medical research, Urology

## Abstract

Arterial hypertension is a cardiovascular disease that leads to important systemic alterations and drastically impairs normal organ function over time. Hypertension affects around 700 million men of reproductive age and hypertensive men present increased risk for reproductive disorders, such as erectile dysfunction. However, the link between arterial hypertension and male reproductive disorders is associative at best. Moreover, many studies have reported associations between decreased male fertility and/or semen quality and alterations to general male health. In this study we aim to investigate the effect of systemic high blood pressure in sperm quality, sperm functional characteristics and testicular physiology in a rat model. Hypertensive rats presented altered testicular morphology – mainly vascular alterations and impaired testicular vasomotion. Hypertensive rats also presented decrease in sperm concentration, DNA integrity and increased percentages of sperm with dysfunctional mitochondria, intracellular superoxide anion activity and abnormal morphology. This study provides mechanistic insights by which arterial hypertension affects the testes, evidencing the testes as another target organ for hypertension as well as its impact on sperm quality.

## Introduction

Recent literature has been amounting demonstrating an almost unequivocal link between human male fertile potential and general male health^1–5^. Many studies have demonstrated a relation between reduction in fertility rates and/or in semen quality and the occurrence of chronic diseases, such as cardiovascular diseases, metabolic alterations, and skin diseases, to name a few^2^.

Modern lifestyle has directly affected individual health^6^. Particularly, arterial hypertension plays an important role because not only is it one of the most common chronic illness worldwide, it is usually asymptomatic and its diagnosis late. Furthermore, around 700 million men at reproductive age are hypertensive^7^. Arterial hypertension can lead to several complications due to the macro- and microstructural and functional changes in organs^8^. A key point is alterations of the local microcirculation, where arteriolar remodeling, vascular tone alterations and vessel density changes occur^8,9^.

It is also noteworthy that the increased prevalence of cardiovascular disease has also seen a decrease in semen quality, although this decrease is still the subject of much debate^10^. However, decreased semen quality has become a global public health concern, because these men present higher mortality rates when compared to men with normal semen quality^1^. Semen quality, therefore, has been suggested to be a biomarker of male health, and many studies have suggested there is a common etiology between impaired overall individual health and reproductive health.

Regarding chronic diseases and male reproductive health, some studies have demonstrated that hypertension is associated with alterations in semen quality, evidenced by abnormal sperm morphology and increased sperm DNA fragmentation^2,11^. Testes were also altered, presenting hyalinization, decreased lumen/wall proportion of intratesticular arterioles, and increased vascular volumetric density in an experimental model of stroke, with accompanying high blood pressure^12–17^.

Mechanistically, it has been well established that the brain, heart, kidneys, and eyes are major target organs for arterial hypertension^7–9^. However, the testes, which are directly associated to sperm quality and male fertility, have not been extensively studied as a target organ. Therefore, in this study we aim to investigate the effect of systemic high blood pressure in sperm quality, sperm functional characteristics, and testicular physiology. We also elucidate vascular mechanisms by which arterial hypertension exerts its effects in the testes.

## Materials and Methods

### Animals

Male ten week-old spontaneously hypertensive rats (SHR) and normotensive Wistar rats were obtained from breeding stock of the SPF (specific-pathogen-free) rat production of the Institute of Biomedical Sciences of University of São Paulo (ICB-USP) (Animal Facility Network) and housed in a temperature-controlled room (22 ± 2 °C) under a 12:12 hour light-dark cycle and *ad libitum* access to tap water and standard chow. The experimental protocol followed the rules from the National Council for the Control of Animal Experimentation, and the study was approved by the Ethics Committee of Animals Use and Care of Universidade de São Paulo (Register Number 134/2016, CEUA-ICB/USP).

### Blood pressure measurement

Baseline systolic blood pressure was determined before the experiments in conscious male twelve-week-old SHR and Wistar rats by an indirect tail-cuff plethysmography (pneumatic transducer, Power Lab 4/S, AD Instruments Pty Ltd) as previously described^18^. Briefly, rats were preheated at 40 °C for 5 min and systolic blood pressure was defined as the moment that a definitive pulse could be detected. The experiment was conducted in an isolated and quiet room. Results were calculated as an average of three consecutives measurements and expressed as millimeters of mercury (mmHg). Care was taken in selecting an appropriate cuff size for each animal. A systolic blood pressure ≥140 mmHg was considered hypertensive, and ≤125 mmhg normotensive. For the other experiments presented in this work, 24–26-week-old male SHR and Wistar rats were used.

### Direct arterial blood pressure

SHR and Wistar rats were anesthetized with an intraperitoneal injection mixture of ketamine (100 mg/kg;i.p) and xylazine (10 mg/kg;i.p) (Agener União, Embu-Guacu, São Paulo, Brazil, and Ceva Santé Animale, Paulinia, Sao Paulo, Brazil, respectively). Then, a heparinized polyethylene catheter (PE-50) was inserted into the right carotid artery and the catheter was exteriorized in the mid-scapular region. After a 24 hour recovery period, systolic blood pressure, diastolic blood pressure, and heart rate were measured in conscious animals by a pressure transducer (Deltran DT-100; Utah Medical Products,United States) and recorded using an interface and software for computer data acquisition (Quad Bridge Amp/PowerLab 4/30; AD Instruments, Melbourne, VIC, Australia). The experiments were conducted in an isolated and quiet room. Mean blood pressure (MAP) was calculated by: MAP = 1/3 × SBP + 2/3 × DBP.

### Histological routine and analysis

The animals of each group were anesthetized with thiopental sodium (Thiopentax™, Cristalia, São Paulo, Brazil), 50 mg/kg;i.p. Testes were removed and weighted to obtain relative weight (g%; organ weight divided by body weight × 100). Then, testes were fixed in modified Davidson’s fluid^19^, dehydrated in ethyl alcohol, clarified in xylol and embedded in paraffin (Paraplast, Labware-Oxford, St. Louis, MO, USA). Slides were mounted using 5-µm-thick cross-sections, and these were stained with hematoxylin and eosin. Slides were analyzed qualitatively using a Zeiss Scope A1-Axio microscope connected to an AxioCam ICc3 camera, and digitalized images were obtained by the image analyzer Axio Vision, version 4.7.2.

### Intravital microscopy

In order to analyze testicular microcirculation *in vivo*, intravital microscopy was carried out as follows.

#### Surgical preparation

Surgery was similar to what was previously reported^20^, with slight modifications. Rats were anesthetized with an intraperitoneal injection of ketamine (113 mg/kg, i.p) and xylazine (7.4 mg/kg, i.p) (Agener União, Embu-Guacu, São Paulo, Brazil, and Ceva Santé Animale, Paulinia, São Paulo, Brazil, respectively). Then, a polyester tube (PE/3, Scientific Commodities, Lake Havasu City, Arizona, USA) was inserted into the femoral vein for FITC-dextran (Sigma-Aldrich, St. Louis, MO, USA) injection in order to allow observation of the blood vessels. Rats were placed then on a special board which allowed temperature control at 37 °C. The left testis was exteriorized and kept moist with a warm (34 °C) Ringer-Locke solution, pH 7.4. The composition of the Ringer-Locke solution in mmol/L was 154.0 NaCl, 5.6 KCl, 2.0 CaCl_2_, 2.0 H_2_O, 6.0 NaHCO_3_, 5.0 glucose, and 0.1 ascorbic acid (Labsynth, Diadema, São Paulo, Brazil). A transparent plastic film was arranged on the tissue to avoid drying during the stabilization time, which lasted 10 min. A camera was incorporated into a trinocular microscope to facilitate observation of the enlarged image (400×) on the computer screen. Images were recorded and a 0.8 mm longitudinal distance objective with 0.65 numerical aperture was used. Measurements of the vessel diameter were performed using a Leica IM50 image manager software (Leica, Heerbrugg, Switzerland). Arterioles were the blood vessels selected for this study, and their diameters ranged from 20 to 30 µm. Microcirculation characteristics remain basically invariant throughout the course of the experiment.

#### Frequency of vasomotion

To evaluate vasomotion, its frequency was measured and expressed as the blood flow stops during the period of one minute (cycles/min). Complete stop of the blood flow was considered in this study, either at baseline or after stimuli with vasoconstrictors such as norepinephrine (1.4 × 10^−7^ M) or angiotensin II (1.4 × 10^−9^ M), topically applied on the tissue (final volume of 30 microliters). Doses were obtained from a previous study^21^. In a set of rats, prazosin (3 × 10^−7^ M), an α1-adrenergic receptor antagonist, or saline were also topically applied one minute after norepinephrine instillation, and frequency of flow stops was counted after prazosin was added.

### Protein expression by western blotting

Testes were dissected and flash frozen in liquid nitrogen. Total protein lysates were prepared by homogenization in lysis buffer containing 10% RIPA Lysis Buffer 10 × (20–188, Merk Millipore, Massachusetts, USA), deionized water, 1 mM PMSF, 10 mM sodium orthovanadate, 100 mM sodium fluoride, 10 mM sodium pyrophosphate and 0.2% protease inhibitor (P8340, Sigma-Aldrich, Missouri, USA). Tissue extracts were centrifuged at 12000 G at 4 °C for 30 minutes and the supernatant was used for quantification of total protein content using the BCA Protein Assay (#23227, Thermo Fisher Scientific, Massachusetts, USA), according to the manufacturer’s protocol. Protein extracts were diluted in Laemmli Sample Buffer (1:1, #1610737, Bio-Rad, São Paulo, Brazil) containing 350 mM dithiothreitol (DTT) and heated in a thermoblock for 5 minutes at 99 °C. Seventy µg of protein from each sample were applied to 10% or 15% polyacrylamide gels and proteins were separated by one-dimensional electrophoresis. Proteins were then transferred to 0.45 µm polyvinylidene difluoride (PVDF) membranes by wet transfer system, and loading was assessed by Ponceau staining. Membranes were then blocked with blocking solution (3% [w:v] bovine serum albumin in TTBS-Tris Buffer pH 7.6 containing 20 mM Tris-HCl, 137 mM NaCl and 0.1% [v:v] Tween 20), for two hours at room temperature, followed by incubation with specific primary antibodies in blocking solution (anti-hypoxia inducible factor-1 alpha [HIF-1α] [1:1000, #3716, Cell Signaling Technology] or anti- vascular endothelial growth factor [VEGF] [1:500, #05-1117, Millipore]) overnight at 4 °C under constant agitation. Membranes were then washed with TTBS (three times, 20 minutes each) at room temperature, and then incubated with appropriate horseradish peroxidase-conjugated secondary antibodies (Rabbit [1:2000, 111-035-144] and Mouse [1:2000, 115-035-166] Jackson ImmunoResearch) in blocking solution for two hours at room temperature under constant agitation. Membranes were revealed by chemiluminescence (Pierce® ECL Western Blotting Substrate, Thermo Scientific, Massachusetts, USA) in a Carestream Molecular Imaging System (Gel Logic 2200 PRO, Carestream Health, New York, USA) and quantified by densitometry using the ImageJ Launcher software (Wayne Rasband, National Institutes of Health, United States).

### Sperm analysis

**Figure 1:**
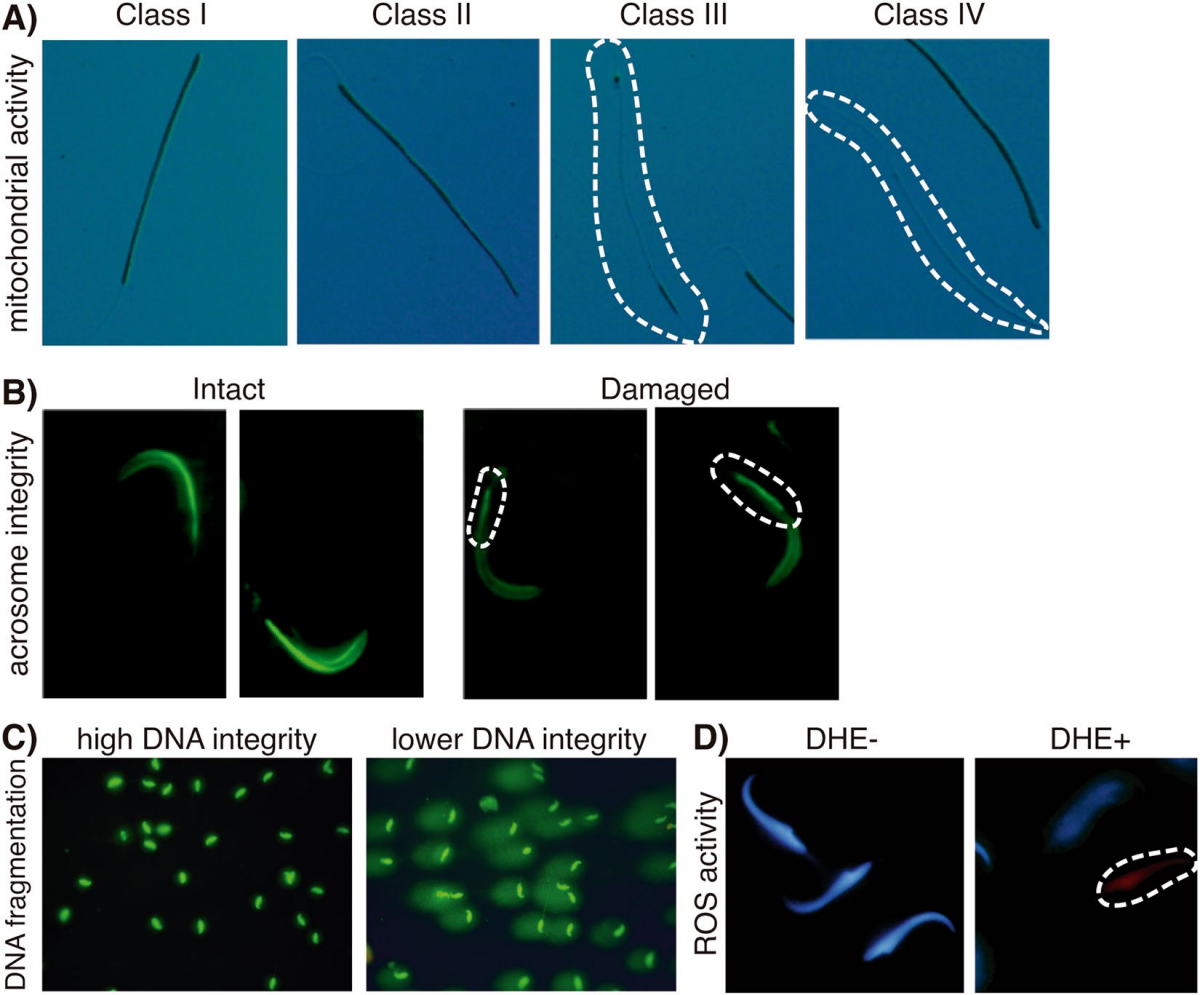
Representative photomicrographs of (**A**) classification of mitochondrial activity in spermatozoa (based on the oxidation, polymerization and deposition of 3,3′-diaminobenzidine by mitochondrial cytochrome c-oxidase): class I - 100% mitochondrial activity; class II - more than 50%; class III - less than 50%; class IV- absence of mitochondrial activity. (**B**) Representative images show the intact and damaged acrosome integrity, assessed using a fluorescent labeled peanut agglutinin (FITC-PNA) that exclusively binds to the outer acrosomal membrane. (**C**) Representative photomicrographs of spermatozoa with high (grade I) and lower (grade II) DNA integrity. Sperm nuclear DNA fragmentation was evaluated by a modified alkaline single-cell gel electrophoresis (comet assay). The comet structure, the “head” refer to the undamaged DNA nucleoid part and the “tail” to the trailing damaged DNA streak. (**D**) Representative images showing negative (blue) and positive (red) dihydroethidium (DHE)-stained nuclei of spermatozoa. DHE is oxidized by reactive oxygen species into ethidium bromide that binds to DNA, emitting red fluorescence.

The animals of each group were anesthetized with thiopental sodium (Thiopentax™, Cristalia, São Paulo, Brazil), 50 mg/kg; i.p Then, the epididymides were removed and the caudal portions of both epididymides from each animal were snipped and immediately immersed in 2 mL of Biggers-Whitten-Whittingham (BWW) media pre-heated to 37 °C. This was incubated for five minutes in order to allow for sperm to flow up into the media, and the epididymis was then removed. Sperm were then used for assessment of sperm concentration, morphology, motility related variables and analysis of sperm functional traits (mitochondrial activity, acrosome integrity, intracellular superoxide anion activity, and DNA integrity) (Fig. 1) and images were examined blind by one trained researcher.

#### Sperm concentration, morphology and motility

For sperm concentration, the samples (spermatozoa diluted in BWW) were diluted 1:20 (vol/vol) in water, and 10 μL were added to each side of a Neubauer chamber. The 25 central squares were used for counting sperm, and the total number of counted sperm was multiplied by 100,000 (to account for chamber height, to transform into concentration per mL, and to account for the initial dilution). If the difference between each side was over 5%, the procedure was repeated, in order to ensure accuracy^22^. The results were presented as million sperm/mL. For morphological analysis of gametes, the smears were stained with hematoxylin-eosin and they were evaluated using a total magnification 1000x with a high qualitative 100x objective. The total of 100 spermatozoa were examined randomly from each animal and classified into two groups: Normal (normotype) and abnormal (teratospermy), according to the criteria for evaluating the morphology of the gamete head and flagellum.

Motility parameters were evaluated using the Computer Assisted Sperm Analysis system (CASA System; IVOS, v. 12, Hamilton Thorn Research, USA). The settings used was previously described by equipment protocol, which were 30 frames with a frame rate of 60 frames/second. Each standard slide (Leja, Netherlands) was pre-heated at 37 °C; 3 μL of sample was placed in the slide. Counting chamber were analyzed with minimum of five fields selected for analysis and 1000 sperms were evaluated per sample. Motility related variables considered were velocity average path (VAP, μm/s), curvilinear velocity (VCL, μm/s), amplitude of lateral head displacement (ALH, μm), beat cross frequency (BCF, Hz), straightness (STR, %) and linearity (LIN, %).

#### Evaluation of mitochondrial activity

In order to evaluate sperm mitochondrial activity, we used the method proposed as previously^23^. This method is based on the oxidation, polymerization and deposition of 3,3′-diaminobenzidine (DAB) by mitochondrial cytochrome c-oxidase.

Briefly, spermatozoa were suspended in a 1 mg/mL solution of DAB in phosphate-buffered saline (PBS, 137 mM NaCl; 2.7 mM KCl; 10 mM Na_2_HPO_4_; 1.8 mM KH_2_PO_4_), in a proportion of 1:1 to 1:5 (vol:vol) semen:stain, depending on sample sperm concentration, and incubated in a water bath at 37 °C in the dark for 1 h. Two 15 μL smears were prepared on conventional glass slides. After drying, the slides were fixed in 10% formaldehyde for 10 minutes at room temperature.

Slides were analyzed in an Olympus BX-51 microscope equipped with phase contrast (Olympus, Japan). A total of 200 cells were counted at 1,000x magnification for each animal. Cells were classified as Class I (100% of the midpiece stained– all mitochondria active), Class II (more than 50% of the midpiece stained), Class III (less than 50% of the midpiece stained), or Class IV (no staining of the midpiece – all mitochondria inactive).

#### Determination of acrosome integrity

Sperm acrosome integrity was assessed using a fluorescent labeled peanut agglutinin (FITC-PNA) (Sigma Aldrich) that exclusively binds to the outer acrosomal membrane^24,25^. Briefly, two aliquots of 15 μL per sample were smeared onto microscope slides, fixed in methanol for 15 minutes, and air-dried. Slides were then stained with 60 µg/mL FITC-PNA in phosphate-buffered saline for 30 minutes in the dark and subsequently washed with Milli-Q water to remove background staining.

For quantitative assessment of acrosome integrity, 200 spermatozoa per sample were counted using an Olympus BX-51 epifluorescence microscope with appropriate filters at 1,000× magnification. Spermatozoa were classified as acrosome damaged or acrosome intact.

#### Determination of intracellular superoxide anion activity

To evaluate the intracellular superoxide anion activity, sperm were incubated with dihydroethidium (DHE - D11347 - Life Technologies, USA), a widely used sensitive superoxide probe. This reagent is oxidized by reactive oxygen species into ethidium bromide that binds to the DNA and then it shifts its fluorescence emission from blue to bright red. Briefly, 10 μL of sperm sample were incubated with 2 μM DHE and 16,23 μM Hoechst 33342 (H33342 - Sigma, St. Louis) both diluted in PBS, at a proportion of 1:1 (vol/vol) semen/stain in the dark at room temperature for 15 minutes. Samples were then smeared onto standard glass slides and two hundred sperm were evaluated using an Olympus BX51 epifluorescence microscope (Olympus, Japan) equipped a wide-pass filter (518 nm–606 nm) and Hoescht (350 nm–461 nm), at 1,000x magnification. The percentage of positive DHE-stained sperm was calculated for each sample^26^.

#### Sperm DNA fragmentation analysis

Sperm nuclear DNA fragmentation was evaluated by a modified alkaline single-cell gel electrophoresis, or Comet assay, as previously performed^27^ with slightly modification. Slides were stained with SYBR Green (SYBR Green II RNA gel stain), diluted 1:10,000 (vol/vol) in TBE (0.1 M Tris [GE Healthcare, Amersham Place, UK]; 0.083 M boric acid; 0.001 M Na_2_- ethylenediaminetetraacetic acid [Carlo Erba Reagents, Cornaredo, Italy]) for 40 minutes, and washed with TBE to remove background staining. A total of 100 sperm were analyzed using an Olympus BX-51 epifluorescence microscope, under 400x magnification magnification and spermatozoa were classified according to the intensity of DNA damage observed by the tail and nuclear fluorescence intensity, divided into grades I (high DNA integrity), II (low DNA fragmentation), III (increased DNA fragmentation), or IV (high DNA fragmentation).

### Statistical analysis

Data were tested (Graph Pad Prism 6.0 Software Inc.San Diego, CA,United States) for normality of distribution using a Kolmogorov-Smirnov test, and for homogeneity of variance using a Levene test. Normally distributed and homoscedastic variables were analyzed by an unpaired Student’s T test or One-way ANOVA. Results are presented as “mean ± standard deviation (SD)”. Statistical significance was set at a maximum alpha error of 5%. For each experiment 4–7 animals were used per group.

## Results

### Characterization of the experimental models: SHR and Wistar rats blood pressures and heart rate

**Figure 2:**
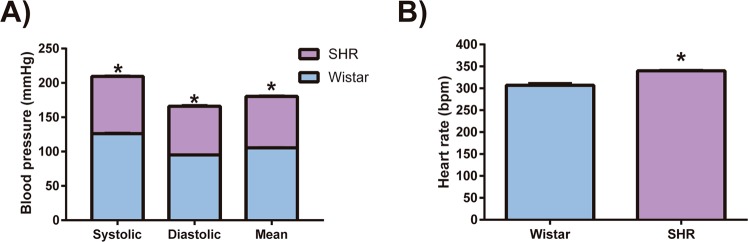
Animal model characterization. (**A**) Systolic, diastolic and mean blood pressure and (**B**) heart rate. Data are expressed as mean ± standard deviation. t-Test student for comparison between groups: *p < 0.05, significant difference vs Wistar rats; n = 5. Spontaneously hypertensive rats (SHR).

Arterial hypertension was defined by reference values of systolic blood pressure of 140 mmHg or above, and/or diastolic pressure of 90 mmHg or above. In this study SHR systolic blood pressure (209.4 ± 2.33 and 126.1 ± 3.06, mmHg, respectively, p < 0.0001, n = 5) and diastolic blood pressure (165.9 ± 3.65 and 95.2 ± 1.59, mmHg, respectively, p < 0.0001, n = 5) were significantly higher when compared to the Wistar rats, resulting in a higher average blood pressure (180.3 ± 2.88 and 105.5 ± 1.83, mmHg, respectively, p < 0.0001, n = 5) (Fig. 2A). Heart rate in conscious animals was also higher in SHR (339.8 ± 4.34, bpm) than in Wistar rats (306.7 ± 11.64, bpm, p < 0.001, n = 5) (Fig. 2B).

### SHR presented morphological alterations in the testicular microvasculature

**Figure 3:**
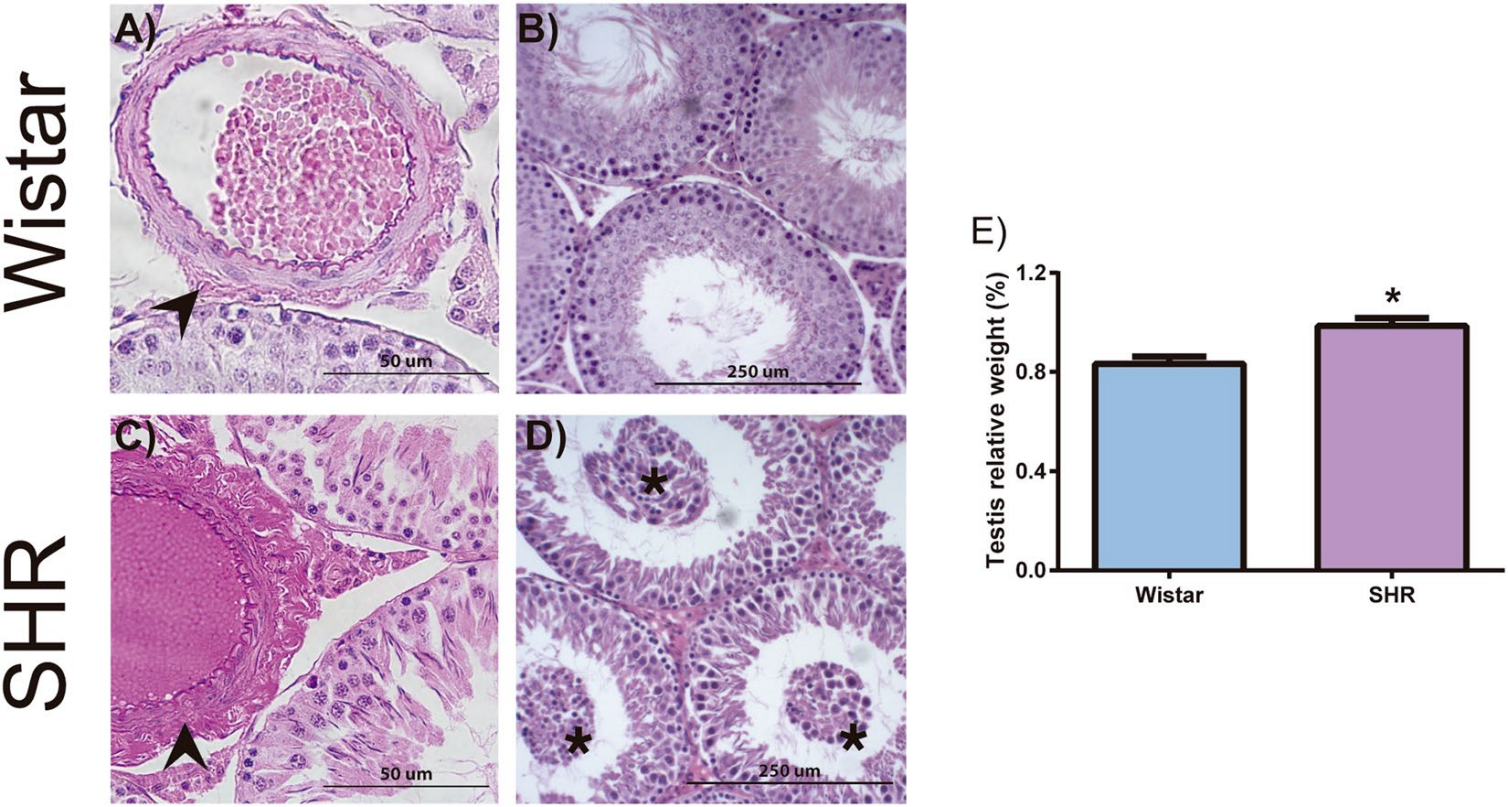
Morphological changes in the testes of SHR. Hematoxylin and eosin stained testicular tissue from 26-week-old control (**A**,**B**; Wistar rats) and hypertensive group (**C**,**D**; SHR). All SHR testes had vascular changes in the arterioles with increased adventitial layer (arrowheads) (**C**). Each rat in hypertensive group shows different stages of testicular damage. Sixty percent of SHR testes show immature cells (asterisk) in tubular lumen (**D**). Hypertensive rats presented increased testicular weight when compared to normotensive rats (**E**). Data are expressed as mean ± standard deviation. t-Test student for comparison between groups: *p < 0.05, significant difference vs Wistar rats; n = 5. Spontaneously hypertensive rats (SHR).

SHR animals presented increased testicular weight when compared to controls (0.98 ± 0.07 and 0.83 ± 0.05, %, respectively, p = 0.01, n = 4–5). Moreover, all SHR animals presented alterations to testicular morphology, both in the interstitial tissue and in the seminiferous tubules (Fig. 3); however, degree of these alterations varied. In the hypertensive group, all animals (n = 5) presented substantial increasing in the adventitial layer of the arterioles (Fig. 3C), independently of their diameter. This result was not found in any of the normotensive Wistar rats. Finally, three of five SHR animals presented seminiferous tubules necrosis or disrupted spermatogenesis, with immature spermatogenic germ cells in the seminiferous lumen (Fig. 3D).

### Impaired testicular vasomotion via α1-adrenergic receptor and increased hypoxia-related proteins of the SHR testis

**Figure 4:**
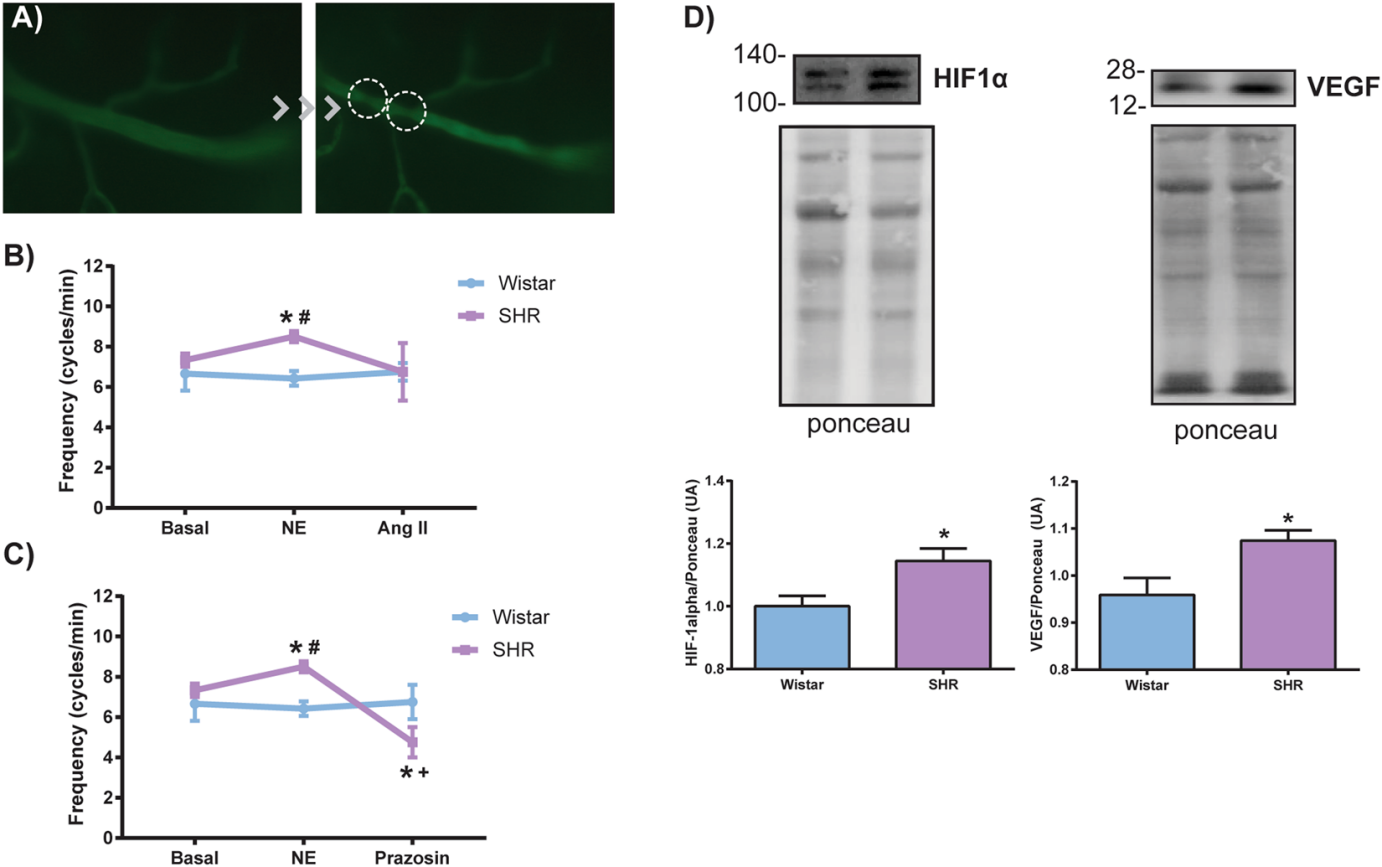
Changes in testicular arterioles blood flow of SHR and protein expression of HIF-1α and VEGF. (**A**) Representative photomicrography of intravital microscopy showing the flow stops in rat testicular arterioles (right). (**B**) The frequency of rhythmic variations in arteriolar blood flow was not altered in SHR at baseline or when stimulated topically with the vasoconstrictor angiotensin II (Ang II), but the stimulation with norepinephrine (NE) increased the blood flow frequency only in the hypertensive group, and (**C**) topical application of prazosin, an α-1 adrenergic receptor antagonist, was able to decrease this frequency. It is important to note that only hypertensive rats responded to the stimulus with norepinephrine and prazosin. (**D**) Representative images of immunoblotting for HIF-1α and VEGF and the respective membranes stained with Ponceau (top). Increased protein expression of HIF-1α and VEGF in SHR testes (bottom). Concentrations: NE: 1.4 × 10^−7^ M; Ang II: 1.4 × 10^−9^ M; Prazosin: 3 × 10^−7^ M. Volume: 30 µL. Data are expressed as mean ± standard deviation. One-way ANOVA or t-test: *p < 0.05 vs Wistar; # vs Wistar NE;^+^vs SHR NE; n = 4–7. Spontaneously hypertensive rats (SHR), hypoxia-inducible factor 1-alpha (HIF-1α), and vascular endothelial growth factor (VEGF).

Because testicular histopathological analyzes demonstrated morphological alterations to the arterioles, we aimed to perform a physiological analysis of testicular microcirculation *in vivo*. No change was observed in the frequency of flow stops in arteriolar circulation between the groups at baseline (7.33 ± 0.51 and 6.66 ± 2.06, cycles/min, SHR and Wistar rats respectively, p > 0.05, n = 6). However, when stimulated with vasoconstrictors, norepinephrine (8.5 ± 0.54 and 6.42 ± 0.97, cycles/min, SHR and Wistar rats respectively, p < 0.01), but not angiotensin II, an increase in the frequency of flow stops was observed only in hypertensive animals (Fig. 4B). Topical application of prazosin, an α1-adrenergic receptor antagonist, was able to decrease (p = 0.01) the frequency of flow stops in these hypertensive animals stimulated with norepinephrine (Fig. 4C) (8.5 ± 0.54 and 4.75 ± 0.95, cycles/min, SHR Ne and SHR prazosin respectively, p < 0.01). Because this alteration could lead to testicular hypoxia, expression of HIF-1α and VEGF was assessed by Western blotting (Fig. 4D). Normotensive Wistar rats presented constitutive testicular protein expression of both HIF-1α and VEGF, while hypertensive rats presented increased testicular protein expression of both HIF-1α and VEGF when compared to Wistar rats (P < 0.05, n = 5).

### Hypertensive rats presented decreased sperm quality

**Figure 5:**
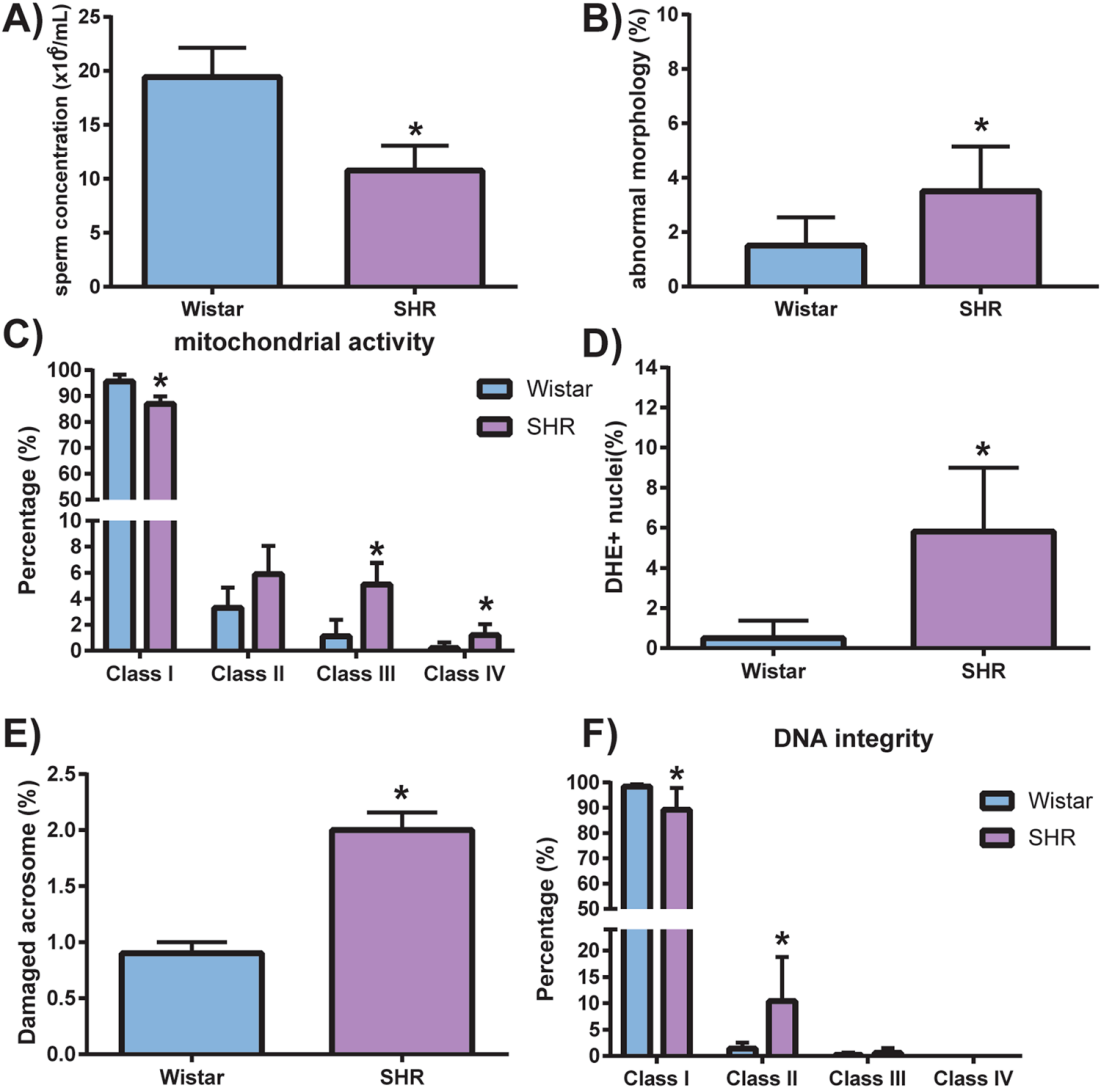
Sperm functional test. SHR had worse seminal quality, evidenced by (**A**) decreased sperm concentration, (**B**) increased percentage of abnormal morphology, (**C**) decreased percentage of mitochondrial activity (DAB classes I, II, III, and IV), (**D**) increased intracellular reactive oxygen species (DHE + nuclei), (**E**) increased acrosome damaged spermatozoa (FITC-PNA-labeled) and (**F**) decreased sperm DNA integrity. Data are expressed as mean ± standard deviation. T-test student for comparison between groups: *p < 0.05, significant difference vs Wistar rats; n = 5–6. Spontaneously hypertensive rats (SHR).

Sperm concentration was decreased by around 44% in SHR when compared to Wistar rats (19.42 ± 6.66 and 10.75 ± 5.699, x 10^6^/mL, respectively, p < 0.05, n = 6) and SHR presented higher percentage of sperm with abnormal morphology when compared to Wistar rats (3.5 ± 1.64 and 1.5 ± 1.04, %, respectively, p < 0.05, n = 6) (Fig. 5A,B). No differences between SHR and Wistar rats were found regarding the analysis of the sperm motility related variables (P > 0.05) (Table 1). SHR animals presented a decreased percentage of sperm with high mitochondrial activity (class I) (87 ± 2.87 and 95.6 ± 2.60, %, SHR and Wistar rats respectively, p < 0.001, n = 5) and increased percentages of sperm with mostly inactive (class III) and with only inactive (class IV) mitochondria, when compared to Wistar rats (Fig. 5C). In addition, hypertensive rats presented higher intracellular superoxide anion activity, as evidenced by an almost 10-fold increase in positive DHE-stained nuclei in the hypertensive group (5.8 ± 3.19 and 0.5 ± 0.86, %, respectively, p < 0.01, n = 5) (Fig. 5D). Likewise, the number of sperms with damaged acrosomes was increased in SHR compared to Wistar rats (2 ± 0.35 and 0.90 ± 0.22%, respectively, p < 0.001, n = 5) (Fig. 5E). Finally, SHR presented decreased percentage of sperm with high DNA integrity (Class I) and increased sperm with initial DNA fragmentation (Class II) (10.4 ± 8.38 and 1.4 ± 1.14, %, SHR and Wistar rats respectively, p < 0.05, n = 5) (Fig. 5F).

**Table 1:** Mean CASA values of spermatozoa in the normotensive (Wistar) and hypertensive (SHR) groups.

	Wistar (n = 5)	SHR (n = 5)	*p*
VAP (µm/s)	91.42 ± 3.37	83.41 ± 20.70	0.417
VCL (µm/s)	166.20 ± 8.54	152 ± 33.51	0.387
ALH (µm)	6.06 ± 0.73	3.93 ± 2.64	0.121
BCF (Hz)	20.04 ± 1.17	19.22 ± 8.09	0.827
STR (%)	57.7 ± 1.14	45.3 ± 14.25	0.08
LIN (%)	33.68 ± 1.08	26.48 ± 8.72	0.104

Data are expressed as mean ± standard deviation; n = 5.

CASA = computer-assisted sperm analysis; SHR: spontaneously hypertensive rats; average path velocity (VAP, µm/s); curvilinear velocity (VCL; µm/s); amplitude of lateral head displacement (ALH, µm); beat cross frequency (BCF, Hz); straightness (STR, %); linearity (LIN, %).

* - statistically significant difference (p < 0.05).

## Discussion

It is alarming how semen quality had decreased in just a few decades and became a major public health concern. It has been suggested that semen quality is a biomarker of man’s overall health. Nowadays, 1.2 billion men are hypertensive worldwide, and it is established that arterial hypertension leads to a multitude of pronounced alterations in organs such as brain, heart, kidneys and eyes, which drastically impairs their normal function over time^8,9^. In this study, we (i) established the testes as another major organ by which hypertension exerts its detrimental effects, (ii) demonstrated an associated vascular mechanism, and (iii) documented detailed alterations in the sperm of hypertensive rats. To the best of our knowledge, this is the first report describing a mechanism by which arterial hypertension affects the testes and important aspects of sperm such as DNA fragmentation, acrosome integrity, mitochondrial and reactive oxygen species activity.

In our study, we used SHR as experimental model of arterial hypertension. SHR have been used in large number of publications related to arterial hypertension, with this model being the most studied (for a review, see^28^). The model arises from breeding of rats with spontaneous high blood pressure. In SHR, blood pressure starts to rise from the 5th week of life, reaching the pressure plateau at week 12, with males having higher values than females^29,30^. In the present study, we used 24–26-week-old rats and all SHR evaluated were hypertensive. Therefore, the hypertensive rats used were exposed for approximately 12 weeks to the plateau of blood pressure. Since the damage of hypertension is due to its mid/long-term effects, further studies investigating the effects during aging would be necessary.

SHR presented increased systolic and diastolic blood pressures, as well as increased heart rate (at rest). Since blood pressure is the product of total peripheral resistance and cardiac output, it has been described that SHR present both altered resistance and conductance vessels in several vascular beds at the age used in this study^31–35^. Moreover, SHR have been shown to present hypertrophy of organs such as heart and kidneys^36^. Here, we showed that hypertensive rats had testicular hypertrophy and this was in agreement with other studies in the literature^37,38^. Suzuki (1979) also demonstrated increased testicular: body mass ratio in SHR stroke-prone (SP) with approximately 21 weeks-old. Interestingly, it was also observed that as the animals were getting older this ratio decreased to the point that values were lower than in normotensive Wistar-Kyoto rats, suggesting that hypertensive damage to the testes might increase with aging^39^.

Aside from testicular hypertrophy, SHR in this study presented alterations in testicular morphology. Most testes from hypertensive animals in our study presented regions with immature germ cells in the tubular lumen as well as tubular necrosis. The unifying factor in all hypertensive rats were the arteriolar changes, especially the increased arteriolar adventitia. High incidence of vascular changes in the testes, brain and kidneys, but not in the ovaries were demonstrated in SHR-SP with up to 71 weeks-old^39^. This could suggest that hypertension might affect more aggressively the microcirculation in male reproductive organs than in females’ reproductive organs. Studies in this experimental model of stroke, which presented severe arterial hypertension, have reported morphological vascular changes in testicular arterioles. In these altered blood vessels, the main observations were also thickening of the intima, fibrinoid necrosis and arteriolar hyalinosis^13,17^. It is known that chronic hypoxia exposure triggers vascular remodeling. The earliest and most influenced structural alterations after hypoxic exposure are found in the adventitial compartment of the vessel wall^40,41^. Corroborating with morphological findings, we also observed for the first time the increased hypoxia-related protein expression of HIF-1α in the testes of hypertensive animals.

It is important to note that the mammalian testes already work on environment with low oxygen levels. This fact is supported mainly for three reasons. First, the partial pressure of oxygen is low. Second, the capacity to increase the blood flow in the testis is also low, because testicular microvasculature is regulated locally and presented vasomotion, a phenomenon that is independent of heart beat. Third, the testicular oxygen consumption is high due the spermatogenesis demands^42,43^. Hypoxia-inducible factor-1 (HIF-1) is a ubiquitously expressed heterodimeric transcription factor that plays a role in oxygen homeostasis and mediates adaptive responses to hypoxia in all nucleated cells^42,44^. HIF-1 presents a subunit O2-regulated denominated HIF-1α and another subunit HIF-1β that are constitutively expressed. In an environment with low oxygen levels, the presence of HIF-1α in the cytoplasm increases, then it translocates into the nucleus and HIF-1α dimerizes with HIF-1β and stimulate the transcription of several genes such as VEGF, erythropoietin, phosphoglycerate kinase-1, and heme oxygenase-1^44,45^. HIF-1 α is constitutively expressed in the rat testes and was increased in periods of testicular ischemia^46^. In the same way, in our study it was observed that even normotensive animals have HIF-1α expression, which was expected since the testes works in a low oxygen environment. More than that, we also observed increased levels of VEGF in hypertensive animals.

The presence of increased levels of HIF-1α suggest that the testes of the hypertensive animals were with lower oxygen levels than normotensive ones. It is also know that one of the primary roles of microcirculation is to ensure adequate oxygen delivery to meet the oxygen demands to the organ^47^. In this way, the testis present unique microvascular features such as testicular vasomotion^48,49^. Testicular vasomotion can be defined as rhythmical and spontaneous contraction and dilatation of arterioles that alters changes in blood flow. It is an important phenomenon that is suggested to control normal function of the testis by acting in the transvascular fluid exchange and as well as the formation of interstitial fluid^48–50^. It is modulated by hormones and altered in several situations, such as varicocele and exposition to smoking. Interestingly, in our study we observed a decreased blood flow, as evidenced by an increased number of flow stops in arterioles after stimulation with norepinephrine, and further demonstrated by recovery after α1 adrenergic receptor antagonism with prazosin, only in hypertensive animals. In another study, Dumber *et al*^48^. observed a decreased testicular blood flow with high doses of norepinephrine and adrenaline when administered into the tail artery of rats. It is known that many stress conditions can lead to increased plasma catecholamines^51^ and that these molecules modulate testicular endocrine function^52–54^. In addition, adult rats submitted to moderate hypoxia presented altered testicular vasomotion and reduced blood flow in the testis^55^.

Many authors have associated general male health to semen quality in several cohorts^1,3–5^. This was further supported by the fact that most testes from hypertensive animals in our study presented morphological alterations. Interestingly, histopathological changes in blood vessels have also been found in testis of infertile and/or men with varicocele^56,57^. It may therefore be the case that cardiovascular diseases in general be another key player regarding alterations in male fertility, which is a possible link to idiopathic male infertility.

In several situations where there are alterations in testicular morphology, there is also altered sperm quality^58,59^, such as decreased sperm concentration and increased abnormal morphology^60^. It is familiar that reduced blood flow could play an important role in the pathogenesis of male infertility^61^. In fact, together with the altered testicular microcirculation we also observed markable alterations in important aspect of sperm in hypertensive rats. Studies in humans have linked hypertension to altered aspects of sperm too. A small cohort study published in 2012 showed that hypertensive men had increased levels of clusterin, a heterodimeric glycoprotein associated with impaired sperm morphology^62^. In 2015, a larger study observed that men with hypertensive disease displayed higher rates of sperm abnormalities^2^. However, neither of these studies showed other aspects such as acrosome integrity, mitochondrial and reactive oxygen species activity or established a possible mechanism.

The intimate relationship between arterial hypertension or male infertility with impaired reactive oxygen species has been demonstrating for several decades. However, this was the first time it has been shown in the literature that hypertension could influence ROS activity in sperm. In our study, the sperm of hypertensive rats presented remarkable increasing in ROS activity. Despite the physiological role of ROS, increased levels can damage cell structures, including lipids and membranes and also lead to alterations in proteins and nucleic acids which could alter their properly function. In fact, we also observed that in addition to ROS, hypertensive rats presented increased DNA fragmentation and number of spermatozoa with damaged acrosome. It was already demonstrated that reduction in testicular blood flow induced an increasing in DNA fragmentation in some germinative cells such as spermatogonia and primary spermatocytes^61^ and damage of sperm DNA can occur at any step of spermatogenesis^63,64^. In other condition with vascular alterations, it was showed that patients with varicocele had reduced testicular arterial blood flow and also increased sperm DNA fragmentation^65,66^. Furthermore, our results demonstrated increased number of sperms with damaged acrosome in hypertensive animals. The acrosome is composed by membranes and proteins, making it sensitive to high concentrations of ROS and to oxidative stress^67,68^. Another convergence point between hypertension and male infertility is regarding to dysfunctional mitochondria. This organelle is known to participate in various cellular functions such as ATP production, steroid hormone biosynthesis, calcium homoeostasis, participate on apoptosis and generation of reactive oxygen species (ROS). In one hand, there is increasing evidence that arterial hypertension is associated with increased mitochondria-derived production of ROS and this was associated with vascular dysfunction^69,70^. In the same hand, it was also suggested that dysfunctional mitochondria in sperm cells could produce oxidants that may contribute to male infertility^71^. In our study, we observed increased percentages of sperm with mostly inactive and with only inactive mitochondria. Similar results were also observed with men that presented varicocele^66^. In fact, it was proposed that mitochondria would be a biomarker of sperm health and fertility^67,72^. These dysfunctional mitochondria in sperm are related to ROS production, especially due to decreased membrane selective permeability, thus allowing efflux of pro-oxidative compounds present within the mitochondria^73^. This might explain the increase in intracellular superoxide anion activity found in our study.

In conclusion, hypertensive rats presented testicular damage associated to alterations in the local microcirculation. This was accompanied by decreased semen quality, increased ROS activity, and increased expression of testicular hypoxia-induced proteins. We also showed a cross-talk between several already known aspects that was altered in both hypertension and decreased male fertility, specially regarding impaired mitochondrial and ROS activity. It is important to focus that this study bring to the light the testis as another important target organ from arterial hypertension. More than that, it also strengthens the link between arterial hypertension and changes in male fertility - a specific case of a link between general male health and fertility. In addition to the role of arterial hypertension in male infertility, we also claim for measure of blood pressure in further human studies of sperm quality, a simple measure that could provide valuable insights to this unexplored field.

## Supplementary information

Supplementary Figure 1.

## Data Availability

The datasets generated during and/or analyzed during the current study are available from the corresponding author on reasonable request.
