# Supplementary material for: Systemic arterial hypertension leads to decreased semen quality and alterations in the testicular microcirculation in rats
Source: Sci Rep. 2019 Jul 30;9:11047. doi: 10.1038/s41598-019-47157-w (PMC6667492; doi:10.1038/s41598-019-47157-w)
Supplement: Supplementary file 1 — Supplementary Figure 1. [file 41598_2019_47157_MOESM1_ESM.pdf]

Systemic arterial hypertension leads to decreased semen quality and alterations in the testicular microcirculation in rats

Lucas Giglio Colli[1]; Larissa Berloffa Belardin[2]; Cinthya Echem[1]; Eliana Hirome Akamine[1]; Mariana Pereira Antoniassi[2]; Rhayza Roberta Andretta[2]; Lucas Solla Mathias[3]; Stephen Fernandes de Paula Rodrigues[1]; Ricardo Pimenta Bertolla[2]; Maria Helena Catelli de Carvalho[1]

[1]Department of Pharmacology, Division of Vascular Biology, Hypertension Section, Instituto de Ciências Biomédicas, Universidade de São Paulo –ICB/USP, São Paulo, SP, Brazil.

[2]Department of Surgery, Division of Urology, Universidade Federal de São Paulo - UNIFESP, São Paulo, Brazil.

[3]Department of Internal Medicine, Botucatu Medical University, Universidade Estadual Paulista - UNESP, Botucatu, São Paulo, Brazil.

Supplementary

Figure

A)

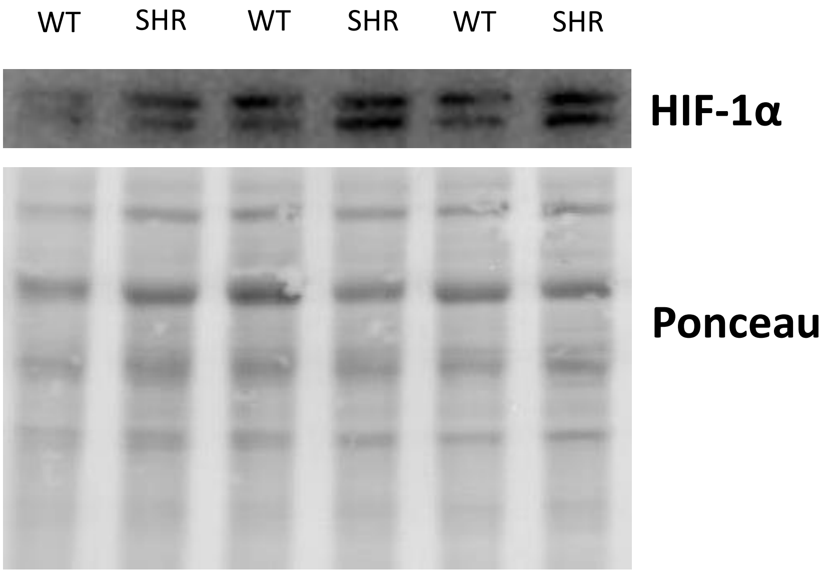

B)

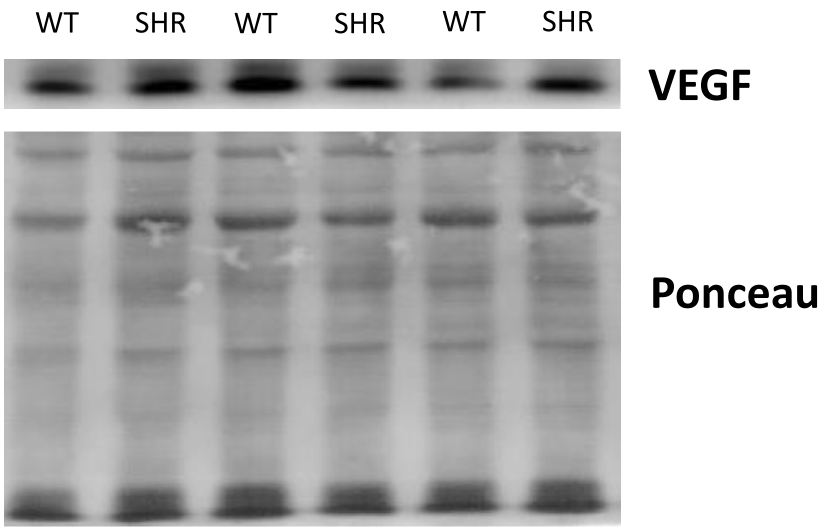

Supplementary Figure 1. Protein expression of **A)** HIF-1α and **B)** VEGF in testes of spontaneously hypertensive rats (SHR) and Wistar rats (WT) by western blot. Hypoxia-inducible factor 1-alpha (HIF-1α), and vascular endothelial growth factor (VEGF).
